# Dynamical analysis of bacteria in microscopy movies

**DOI:** 10.1371/journal.pone.0217823

**Published:** 2019-06-06

**Authors:** Teun Vissers, Nick Koumakis, Michiel Hermes, Aidan T. Brown, Jana Schwarz-Linek, Angela Dawson, Wilson C. K. Poon

**Affiliations:** 1 SUPA and School of Physics & Astronomy, The University of Edinburgh, Peter Guthrie Tait Road, Edinburgh EH9 3FD, Scotland, United Kingdom; 2 Department of Physics, Soft Condensed Matter, Debye Institute for Nanomaterials Science, Utrecht University, Princetonplein 5, 3584 CC Utrecht, Netherlands; Weizmann Institute of Science, ISRAEL

## Abstract

Recent advances in microscopy, computing power and image processing have enabled the analysis of ever larger datasets of movies of microorganisms to study their behaviour. However, techniques for analysing the dynamics of individual cells from such datasets are not yet widely available in the public domain. We recently demonstrated significant phenotypic heterogeneity in the adhesion of *Escherichia coli* bacteria to glass surfaces using a new method for the high-throughput analysis of video microscopy data. Here, we present an in-depth analysis of this method and its limitations, and make public our algorithms for following the positions and orientations of individual rod-shaped bacteria from time-series of 2D images to reconstruct their trajectories and characterise their dynamics. We demonstrate in detail how to use these algorithms to identify different types of adhesive dynamics within a clonal population of bacteria sedimenting onto a surface. The effects of measurement errors in cell positions and of limited trajectory durations on our results are discussed.

## Introduction

The ability of microbes to move on and adhere to surfaces is an essential part of their survival strategies [1]. Motility allows them to explore new niches and swim towards nutrients or oxygen [2, 3] to optimise growth and division. Adhesion allows them to colonise surfaces and grow protective biofilms [4, 5]. In this state, bacteria are typically more difficult to dislodge by fluid flows and can enjoy increased resistance to antimicrobials [6, 7]. As a result, they become sources of infection that are hard to eradicate [8].

Surface colonisation typically starts with the adhesion of a few individual microorganisms, so that it is important to understand the physics of single-cell interaction with substrates. Recently, we studied the adhesion of individual rod-shaped *Escherichia coli* (*E. coli*) bacteria on glass, and discovered significant heterogeneity in adhesive propensity and post-adhesion dynamics amongst a clonal (and therefore genetically identical) population [9]. These results, which have important implications for the design of surfaces to minimise microbial adhesion, were obtained using relatively unsophisticated time-lapsed optical microscopy in 2D coupled with a bespoke suite of image analysis software. Our algorithms successively (1) identify accurately and reproducibly rod-shaped bacteria, (2) track all the individual bacteria in time, and (3) analyse the dynamics of each individual cell on the surface to characterise its behaviour. Although a variety of methods for the individual steps already exist in the literature, our work successfully combined, modified and developed them to allow the high-throughput, semi-automated generation of large data sets.

These methods and their integration into a high-throughput suite were outlined in a recent paper focussing on describing the results we obtained using this methodology. In this paper, we analyse these methodological developments in depth, including possible pitfalls, and carry out a detailed estimation of uncertainties. We also compare our methods with existing algorithms. To achieve these goals, we use data obtained in the same way as in Ref. [9], but from an entirely independent set of experiments. At the same time, we are releasing our software publicly on GitLab. Taken together, what we present here should enable others to adapt our methodology to study the adhesion of other microorganisms, or to dynamical studies of microbes unconnected with adhesion. Indeed, we believe that our methodology should prove useful for studying the dynamics of anisotropic synthetic colloids where significant polydispersities in shape, size and behaviour exist.

## Materials and methods

### Sample preparation

*E. coli* bacteria were grown on Lysogeny broth (LB) agar plates. An inoculated colony was transferred to 10 ml of liquid LB, which was then incubated overnight for 16 h at 30 °C. From this, a fresh culture was inoculated at a 1:100 dilution in 35 mL of tryptone broth and grown for 4 hours to late exponential phase. Next, the cells were washed by careful filtration three times with motility buffer (MB) [MB: aqueous solution containing 6.2 mM K_2_HPO_4_ (Sigma-Aldrich), 3.8 mM KH_2_PO_4_ (Fisher Chemical), 67 mM NaCl (Fisher Chemical), 0.1 mM EDTA (Sigma-Aldrich)] and redispersed in MB with 0.72 μM glucose. The final optical density just before the start of the experiment was OD ≈ 0.03 (λ = 600 nm) corresponding to a concentration *C* ≈ 4.5 × 10^7^ cells/mL [10].

We used *E. coli* strain AB1157 WT [11] and strain AD31 (MG1655 fliF,fimA = ΔFF_MG_)) with genes coding for flagella and fimbrae deleted. Strain AD31 was constructed by P1 transduction from strain JW4277 (BW25113 fimA) [12] into strain AD26 (MG1655 fliF) [9], following removal of the kanamycin resistance cassette in FliF using flp recombinase expressed from plasmid pCP20.

### Microscopy and optics

We used a Nikon TE300 Eclipse inverted microscope with a 60× Ph2 objective and focused in an optical plane just above the capillary surface so that bacteria on the surface appeared dark against a bright background. To record movies, we used a Mikrotron MC 1362 high-speed camera. The dispersion of bacteria in MB was injected gently with a pipette into a borosilicate glass capillary (Vitrocom, 0.4 mm × 8.0 mm × 50 mm), which was then sealed with Vaseline and placed on a motorised stage. Multiple locations on the lower glass surface of the capillary were followed in time and a movie was recorded when a position was visited. Each movie 1008 frames recorded at 30 frames/s at a resolution of 1040 × 1024 pixels.

### Data analysis

Positions and orientations of individual cells were determined from each image frame in the recorded movies. Trajectories were constructed by identifying corresponding (typically the nearest) cells in consecutive frames. Subsequently, trajectories were filtered by removing the sections where two cells were too close together. The mean-squared displacement (MSD) and mean-squared orientational displacement (MSOD) as functions of delay time were then calculated for each trajectory, from which translational and orientational exponents were calculated to characterise cell dynamics.

Datasets used for this work are publicly available (Edinburgh DataShare [13]). At the time of publication, software repositories to determine positions and orientations of rods (*findRods2Dt* [14]), construct trajectories (*trackRods2Dt* [15]), filter trajectories (*filterTracks2Dt* [16]) and analyse trajectories (*analyzeBugTracks2Dt* [17]) are available on GitLab under the GNU General Public License v3.0. The code is written in *C* for Linux. We also added *Python* scripts to read positions and trajectories [18]. To ensure long-term availability, a copy of the code in its current form is also added to the data repository [13].

## Results and validation of algorithms

### Identifying bacteria

To follow the dynamics of individual *E. coli* cells on the surface, it is required to extract their positions and orientations within recorded movies. In our 1040 × 1024 pixel^2^ tiff-images at 60× magnification one pixel corresponds to 0.234 × 0.234 μm^2^. A 2 μm × 1 μm spherocylindrical *E. coli* bacterium appears as an ≈ 10 × 5 pixel^2^ rod. Typical movies contained 1008 frames (tiff-images), and we recorded several hundreds of movies for an individual experiment.

There is a wide variety of software to recognise particles or cells from images. Some software even focuses on various complex shapes [19] or is designed specifically to study individual cells within growing colonies [20], even in 3D and in dense systems using confocal microscopy [21], or super-resolution microscopy and powerful segmenting strategies [22].

As before [9], our objective here was to study differences between bacteria on the surface in the dilute case where there is ample distance between individual cells that all have a similar rod-like shape. Although there was thus no need to separate partially overlapping cells or deal with irregular shapes, the algorithm had to quickly identify cells in a robust way for large numbers of recorded movies. To this end, we adapted an earlier algorithm [23] to find colloidal rods in 3D from confocal microscopy images. We modified it to find rod-shaped objects representing bacteria for 2D-images in our datasets in a high-throughput manner that is robust and minimises the number of both false negatives (does not find a bacterium where there is one) and false positives (identifies a bacterium in a place where there is none). The program, *findRods2Dt* [14], for which a detailed code-based description is also available in its repository, works as follows.

**Fig 1:**
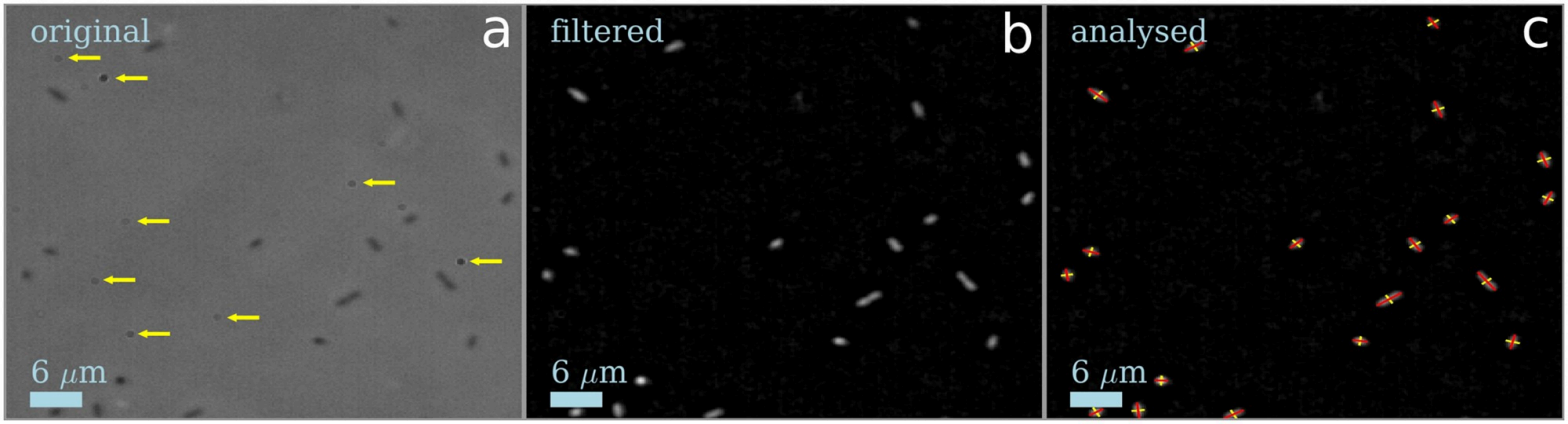
Identifying bacteria on surface. **(a)** Part of an original image as obtained with phase contrast microscopy, dark signal indicates objects that are in focus. The yellow arrows indicate artefacts produced by dust in the optical path, here from the glass cover protecting the camera chip. **(b)** The same image after artefact removal, black/white inversion, Gaussian blurring, local background subtraction and intensity renormalisation. **(c)** Bacteria with their long (red) and short (yellow) axes determined.

We first filter out noise and background artefacts. The latter can arise, e.g., from dust particles on the glass covering of the charge coupled device (CCD) of the camera. Fig 1a shows part of a raw image with the artefacts indicated. To filter these out, we averaged randomly selected multiple images recorded at different locations in the capillary, and identify the artefacts, which do not change from image to image, as spots with particularly high or low intensities; these are then subtracted, Fig 1b. In the software, parameters such as background intensities to subtract, approximate width of bacteria, as well as filtering and other settings can be entered manually.

We next use a Gaussian blurring kernel (standard deviation *σ* ≈ 0.7 pixels) to smooth out the noise. Remaining background inhomogeneities on the tens of pixels scale arising from out-of-focus cells were removed by convolving with a top-hat kernel with diameter ∼ 20 pixels and subtracting this from the unconvolved image. In other words, for each pixel, the average background in a square of the surrounding 20 pixels is subtracted from its intensity. Finally, we remove bright pixels that are outliers and then renormalise the signal to give intensities between 0 and 1. After these steps, each bacterium appears as a bright groups of pixels against a relatively uniform background, Fig 1b.

Identified bacteria are shown in Fig 1c. To find the positions and orientations of individual cells, rod-shaped ‘islands’ of sufficiently high intensity are identified. Following previous work that is already described in detail [23], we search for backbones, straight lines of bright pixels on a local maximum or a saddle point. The pixels within one radius of all sufficiently bright backbones are then grouped into islands. Next, the algorithm calculates the co-variance matrix of each island and uses its eigenvectors to determine the orientations and approximate lengths of the long axis (eigenvector with the larger eigenvalue) and short axis (eigenvector with smallest eigenvalue), as well as an approximate centre of mass (CM), taken to be the weighted position-average of pixel intensities. To refine the CM, the positions of the poles are determined by calculating the average intensity along the long axis and then the poles are taken to be the the points on either side where the intensity becomes half of this average value. The width is determined in a similar way. Now, the refined CM is calculated as the point halfway between both poles in the length direction. We find that this procedure gives stable results, even when there is an intensity gradient along the long axis (e.g. if the bacterium is tilted slightly out-of-plane) or if there are background artefacts.

We have validated the code using images obtained via phase contrast microscopy but our method is sufficiently general that it should be applicable to other techniques, e.g. epifluoresent, confocal or dark field microscopy.

### Tracking bacteria

**Fig 2:**
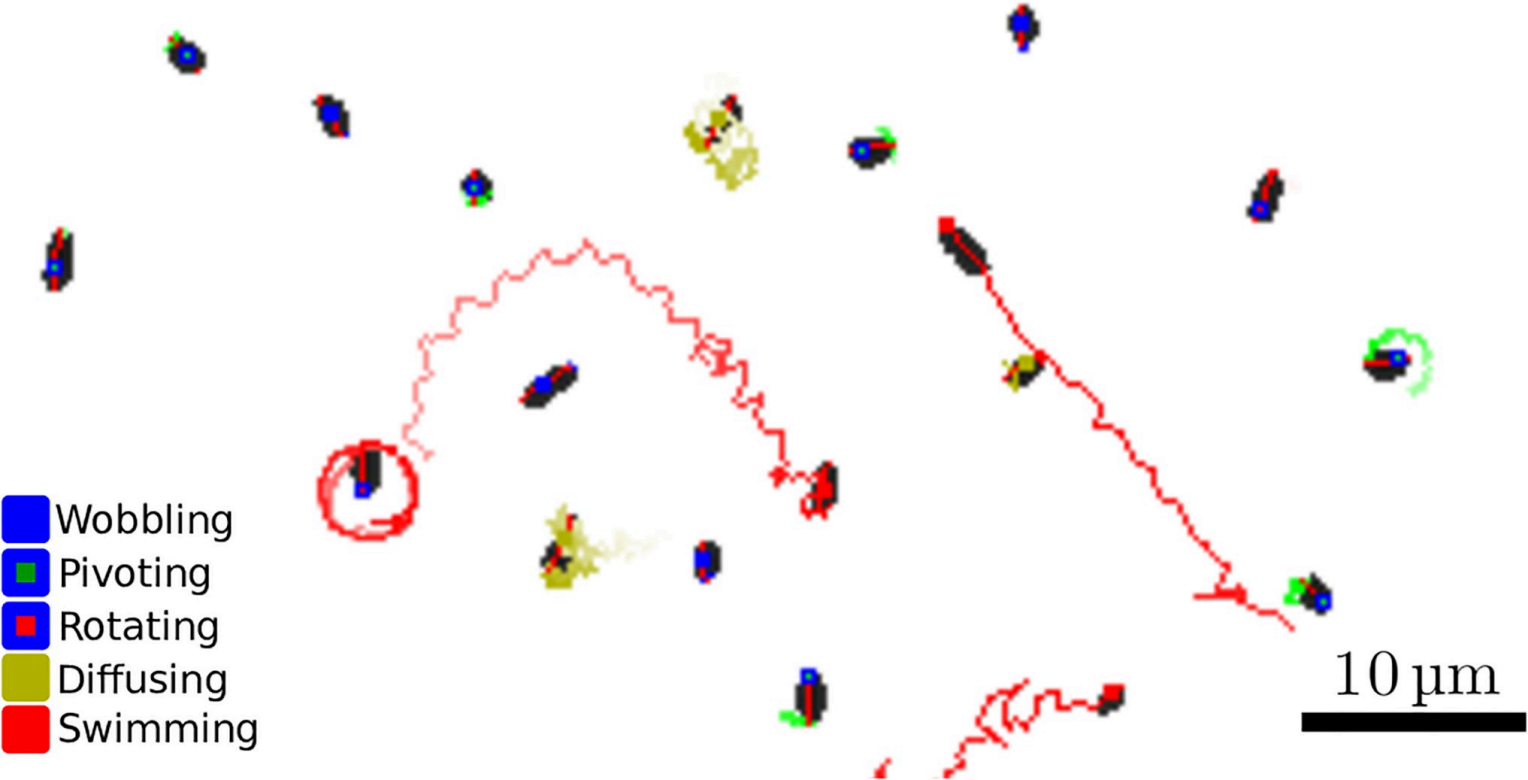
Trajectories of bacteria on the surface. The image is black/white-inverted (w.r.t. the filtered image in Fig 1b) and thresholded to be clear in print. For each cell in the image, the point of minimal motion (anchoring point for adherers) is marked with a square. The combination of the inner and outer color of the square denotes the cell type (see legend). For swimmers and diffusers, the traces show the trajectory of the point of minimal motion. For adherers the trace shows the trajectory at the cell’s point of maximum motion (one of its poles) to visualise pivoting or active rotation dynamics.

To characterise the dynamics of individual cells, we employed an algorithm *trackRods2Dt* [15] that calculates trajectories from the determined positions in consecutive frames (Fig 2). For each bacterium in each frame, a list of nearest neighbours in the next frame is made, from which a possible match is found. By repeating this for consecutive frames, trajectories are constructed. To correctly track the faster-swimming bacteria, we use each displacement as a prediction for the next and look around this position for possible candidates. For diffusing bacteria, such a prediction will be correct in only half the cases (diffusion is random, so there is an equal probability a bacterium moves in one direction or the other). The tracking algorithm does not yet take into account whether a bacterium is diffusing or swimming. This is not a problem in our case, however, since the displacements involved for diffusing cells are small and the correct candidates are still found. Even for dilute dispersions, it sometimes happens that two or more trajectories intersect. We devised a separate program, *filterTracks2Dt* [16], to deal with this by removing segments within a trajectory where cells are within close range of others, and create two new trajectories from the remaining segments on either end of it.

### Dynamical analysis of bacteria

In our code to analyse trajectories *analyzeBugTracks2Dt* [17], we determine the mean-squared displacements along the length axis of each cell within a trajectory, calculate the translational and rotational exponents, and use these parameters to categorise the cellular surface dynamics. Specific microscope parameters such as the frame rate, pixel size can be entered manually. For our analysis we only consider trajectories with a duration of at least 25 frames (∼ 0.8).

#### Determining the anchoring point of adhering cells

**Fig 3:**
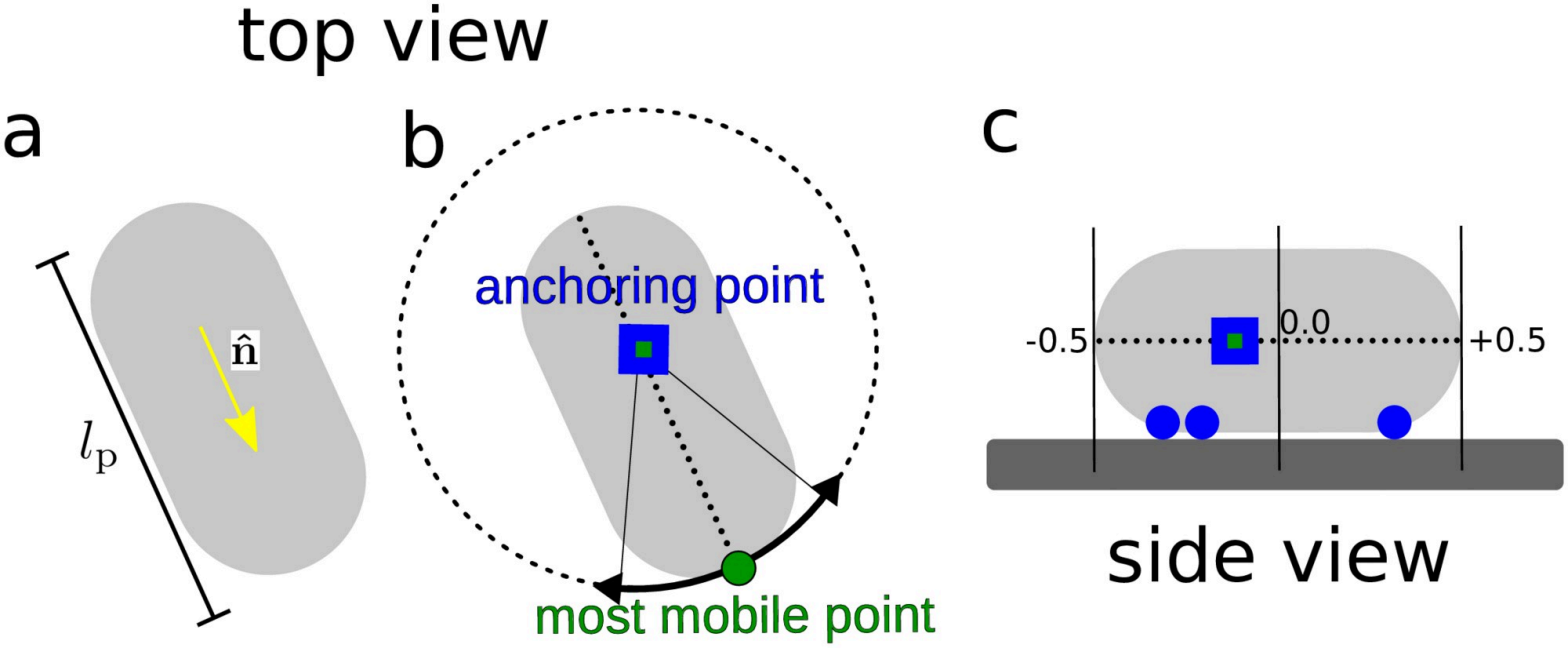
Schematic of an adhering cell. **(a)** showing the long axis and orientation, **(b)** anchoring point (least mobile point), and the most mobile point, and **(c)** side view showing an example where a cell is stuck at three adhesive spots (blue circles), the anchoring point (blue square with green centre) being the average of those; the numbers show the fractional coordinates *f* ∈ [−0.5, 0.5], where *f* = 0 at the centre and *f* = ±0.5 at the poles.

To characterise the dynamics of individual bacteria, we identify the point of minimal motion along the length axis of each individual cell within a trajectory [9]. For each bacterium *i* with time-dependent position **r**_*i*_(*t*), we calculate the mean-squared displacement (MSD) $\langle R_{i}^{2}\left(f,\tau =0.4s\right)\rangle_{t}=\langle {\left(r_{i}\left(f,t+\tau \right)-r_{i}\left(f,t\right)\right)}^{2}\rangle$ at 31 different points $r_{i}\left(f,t\right)=r_{i}\left(0,t\right)+f_{i}{\hat{n}}_{i}\left(t\right)l_{i}^{p}\left(t\right)$ along the long axis, where *f*_*i*_ ∈ [−0.5, 0.5] is the relative dimensionless coordinate along this axis, $\hat{n}$ and $l_{i}^{p}\left(t\right)$ the normalised orientation vector and projected length of the bacterium at time *t* (Fig 3a), and *τ* = 0.4 s is the delay time over which the MSD is measured. This relatively short delay time ensures sufficient statistics is available even for the shorter trajectories amongst those considered for analysis.

Finally, we determine the point of minimum MSD. For diffusing cells this point of minimal motion roughly corresponds to the hydrodynamic centre. For adhering cells, we refer to the point of minimal motion as its ‘anchoring point’. For a cell adhering at a single spot, this point roughly corresponds to the point of attachment. For cells adhering at multiple spots, the anchoring point represents a weighted average of these spots. A schematic of an adhering cell and its anchoring point is shown in Fig 3b and 3c.

#### Characterising dynamics using translational and rotational exponents

There are many ways to characterise the dynamics of individual cells. In the simplest method, adhering cells can be identified by recording images at long shutter times, so that moving cells are smeared out and adhering cells appear as a single spot [24, 25]. Displacements or MSDs from movies can be used, where adhering cells have the lowest, and diffusing and swimming cells show larger displacements [26, 27]. Alternatively, other parameters are used to distinguish different dynamics, such as the curvature and directionality of the trajectories [28] or by looking at relative angles between consecutive displacements for different temporal coarse-grainings [29].

We distinguish between different types of dynamics in individual cells by using their translational and rotational power-law exponents [9]. To calculate these, we use values of the MSD(*τ*) and MSOD(*τ*) at different delay times *τ*. This has the advantages of not requiring *a priori* knowledge of typical values of the MSD for different kinds of dynamical behaviour. Instead, the calculated exponents will always fall in a well-defined range independent of sample-specific details such as diffusion constant or mean swimming speed.

**Fig 4:**
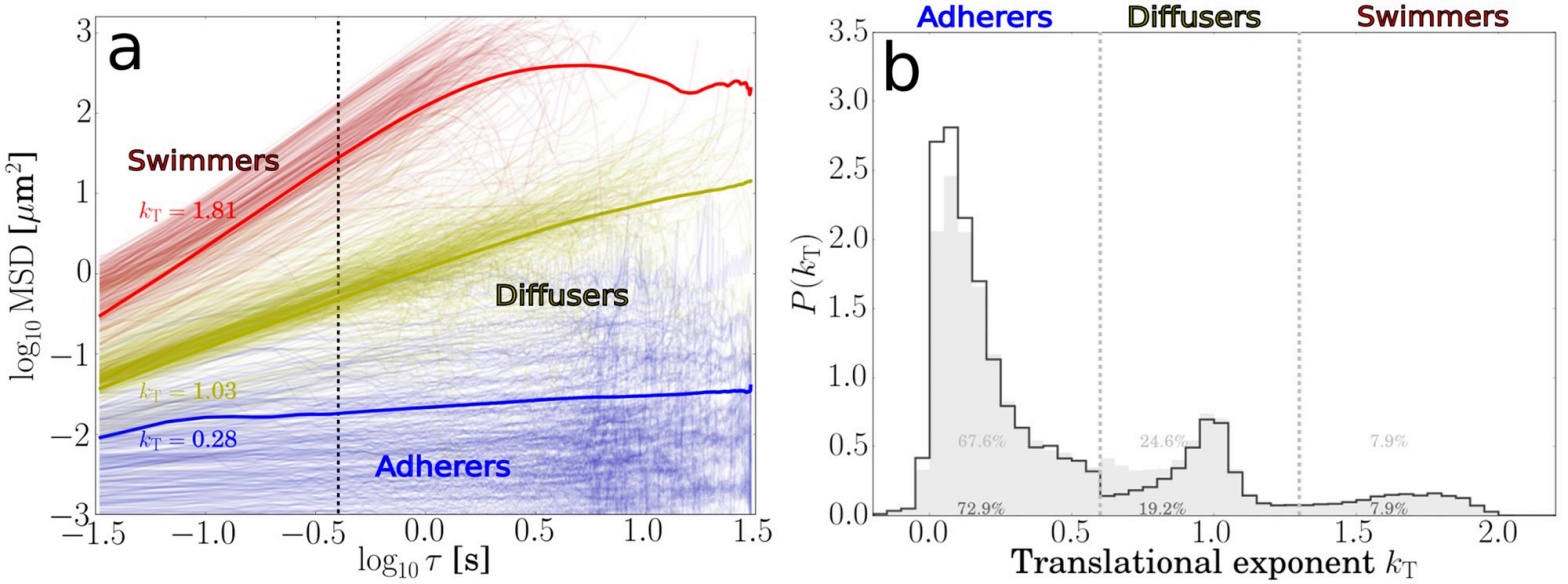
Calculation and distribution of translational exponents used to identify adhering, diffusing and swimming cells. **(a)** MSD of the anchoring point versus delay time on a log-log plot for adhering, diffusing and swimming bacteria. The slope gives the translational exponent, the vertical dotted line marks the delay time *τ* = 0.4s. Thin lines represent data from individual trajectories. Thick lines represent averages for each category and are calculated from duration-weighted trajectories. **(b)** Distribution of translational exponents showing peaks for adhering, diffusing and swimming cells. The gray distributions represent exponents calculated for shorter delay times (*τ* = 0.4s), black lines are for distributions including exponents calculated for longer delay times (*τ* = 4s).

The translational exponent *k*_T_ is calculated as follows:

$$\begin{array}{c} k_{T}=\frac{\text{log}\left(\langle R^{2}\left(\tau_{1}\right)\rangle \right)-\text{log}\left(\langle R^{2}\left(\tau_{0}\right)\rangle \right)}{\text{log}\left(\tau_{1}\right)-\text{log}\left(\tau_{0}\right)}, \end{array}$$ (1)

where *τ*_1_ = *τ* is the delay time over which the MSD is measured, and *τ*_0_ = 1/framerate is the delay time between two consecutive frames. Adhering cells show *k*_T_ ≈ 0, diffusing cells show a distribution around *k*_T_ ≈ 1, while swimming cells show *k*_T_ ≈ 2 (Fig 4a).

The minimum duration of a trajectory to be considered for analysis is 25 frames (∼ 0.8). Therefore, we calculated the translational exponents $k_{T}^{0.4s}$ using a delay time *τ* = 0.4s, shorter than the minimum trajectory duration. The distribution of translational exponents for *τ* = 0.4s is shown in Fig 4b (gray area), showing distinct peaks for adhering, diffusing and swimming cells. We identified swimming cells for $k_{T}^{0.4s}>1.3$, diffusing cells for $0.6<k_{T}^{0.4s}<1.3$ and adhering cells for $k_{T}^{0.4s}<0.6$.

Occasionally, it occurs that adhering cells can appear as swimming or diffusing on this short time-scale, as their short-term dynamics has a diffusive or ballistic component. To deal with this, we used two additional rules. First, we demanded that trajectories of adhering cells have a duration of at least 6s. Trajectories with a duration less than this but $k_{T}^{0.4s}<0.6$ were identified as ambiguous. For trajectories with a duration longer than 6s but $k_{T}^{0.4s}>0.6$, we also calculated the translational exponent $k_{T}^{4s}$ for a longer delay time *τ* = 4s. A trajectory for which $k_{T}^{4s}<0.6$ is identified as adhering, even if $k_{T}^{0.4s}>0.6$. The result is that cells otherwise detected as diffusing are now correctly identified as adhering. The distribution using this additional rule is shown in Fig 4b (black line) and shows a slightly better separation between the adhering and diffusing peak.

**Fig 5:**
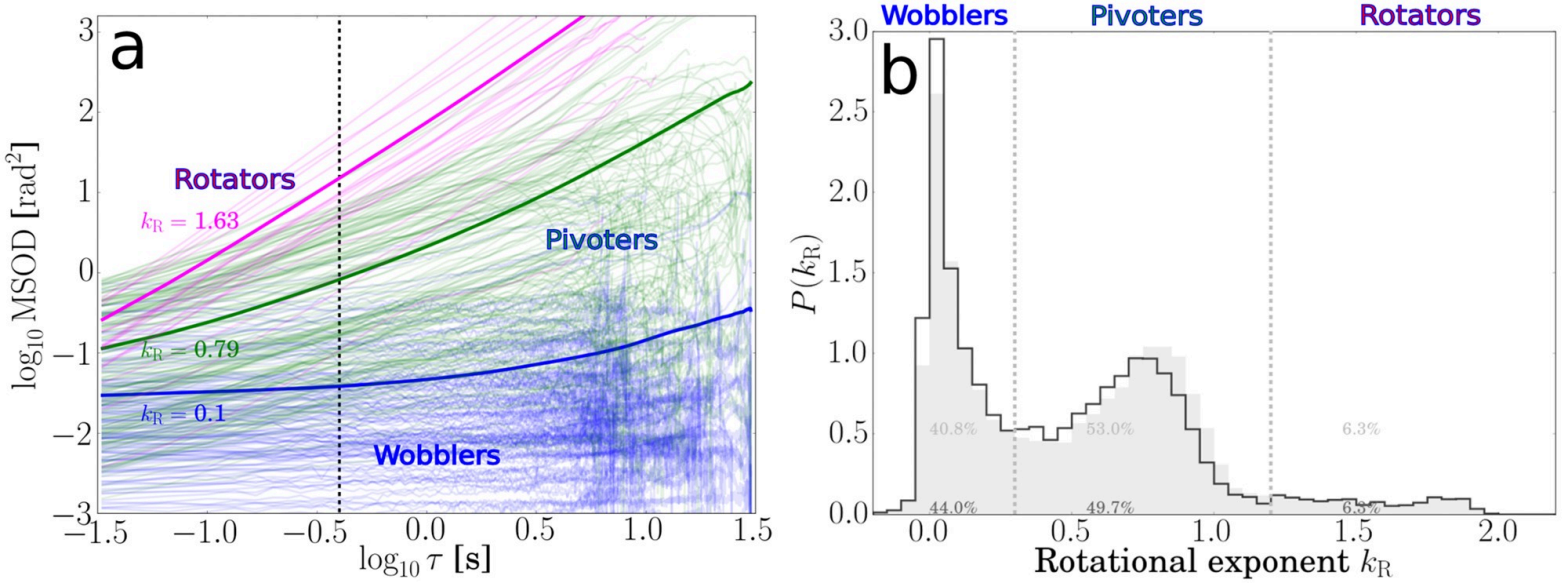
Calculation and distribution of rotational exponents used to identify different post-adhesion dynamics. **(a)** log-log plot of the MSOD versus delay time for adhering bacteria reveals different post-adhesion dynamics of cells: wobbling, pivoting or actively rotating. The slope gives the rotational exponent, the vertical dotted line marks the delay time *τ* = 0.4s. Thin lines represent data from individual trajectories. Thick lines represent averages for each category and are calculated from duration-weighted trajectories. **(b)** distribution of rotational exponents for adhering cells showing regions for wobbling, pivoting and rotating post-adhesion dynamics. The gray distributions represent exponents calculated for shorter delay times (*τ* = 0.4s), black lines are distributions including exponents calculated for longer delay times (*τ* = 4s).

Once the adhering cells are identified, their post-adhesion dynamics can be classified by the rotational exponent

$$\begin{array}{c} k_{R}=\frac{\text{log}\left(\langle \theta^{2}\left(\tau_{1}\right)\rangle \right)-\text{log}\left(\langle \theta^{2}\left(\tau_{0}\right)\rangle \right)}{\text{log}\left(\tau_{1}\right)-\text{log}\left(\tau_{0}\right)}, \end{array}$$ (2)

where 〈*θ*^2^(*τ*)〉 is the mean-squared orientational displacement. Wobblers show *k*_R_ ≈ 0, pivoters *k*_R_ ≈ 1 and active rotators *k*_R_ ≈ 2 (Fig 5a). The distribution of rotational exponents for *τ* = 0.4s is shown in Fig 5b (gray area). We identify wobblers as $k_{R}^{0.4s}<0.6$, pivoters as $0.6<k_{R}^{0.4s}<1.2$, and active rotators with $k_{R}^{0.4s}>1.2$.

Using long-term rotational exponents (*τ* = 4s) in a similar way as the translational ones slightly shifts peaks for pivoters to lower exponents, but does not appear to improve separation between wobblers and pivoters (black line in Fig 5b).

#### Distribution of anchoring points for adhering cells

**Fig 6:**
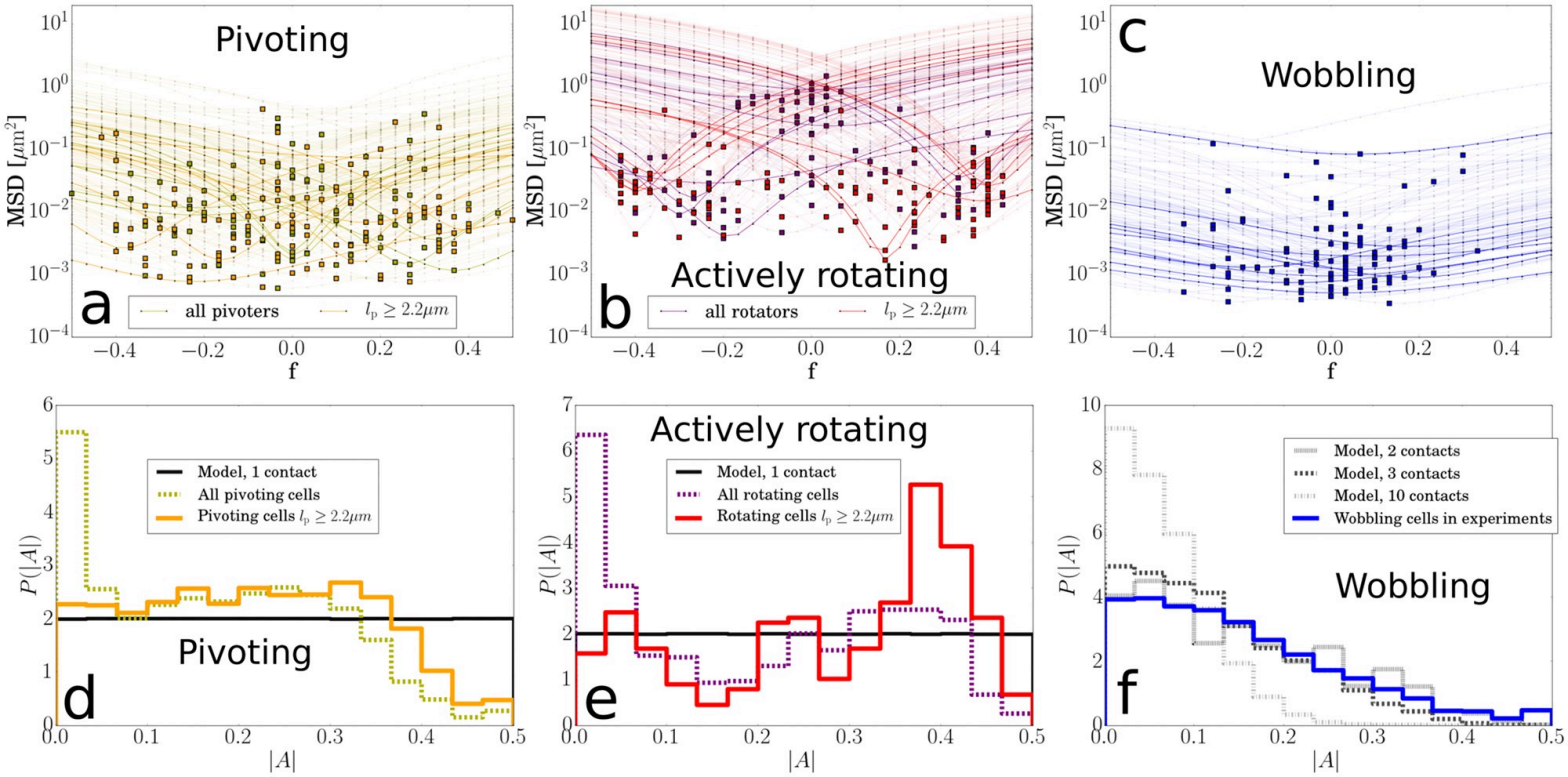
Anchoring points for different types of adhering. **(a-c)** MSD along the length axis for trajectories of 200 pivoters, 200 rotators, and 100 wobblers. For pivoters and rotators, data is shown for 100 cells of any length and for 100 cells with a projected length *l*_p_ ≥ 2.2 μm. For clarity, all but 10 lines are ghosted for each set of examples. For each trajectory, the minimum in MSD denotes the anchoring point. **(d-f)** The distribution of anchoring points along the length axis *A* ∈ [−0.5, 0.5]. The absolute value |*A*| is used because the two poles are taken to be indistinguishable. The histograms are constructed from trajectories of 9404 pivoters (d), 700 rotators (e) and 7415 wobblers (f).

Fig 6a–6c shows the MSD for 31 different points along the length axis for pivoters (a), active rotators (b) and wobblers (c). Each trajectory is represented by a line showing the MSD at each of these points. The lowest MSD value is found at the anchoring point, whereas the highest MSD values are always located at either one of the poles (*f* = −0.5 or *f* = 0.5).

The anchoring point, where the MSD is minimal, is marked with a square. Corresponding distributions showing the density of anchoring points along the length axis are shown in Fig 6d–6f. Note that the absolute value |*A*| is plotted here, because the two poles of a cell are indistinguishable in our images. The distributions of |*A*| for wobbling and pivoting bacteria are similar to those already published [9].

Pivoters adhere at a single adhesive patch along the cell axis, for which the distribution is close to being uniform (Fig 6d). The peak close to the centre (|*A*| = 0) and the trough near the poles (|*A*| = 0.5) are related to artefacts resulting from the spherocylindrical shape of the cells: when only longer cells are selected (it is clearly visible for *l*_p_ ≥ 2.2 μm) the peak disappears. This is also the case for actively rotating cells (Fig 6e).

To explain these artefacts, consider a pivoter or rotator adhering at one of its poles and standing vertical with respect to the substrate; its projection would appear effectively as a spherical cell adhering somewhere near the centre, and therefore with an anchoring point |*A*| ≈ 0. Selecting only cells with a projected length greater than the cell width effectively picks out cells oriented parallel to the surface for which the projected image is close to the real image, removing the peak around |*A*| = 0. Because of the spherocylindrical shape, sticking exactly at either poles would mean the cell is oriented vertical with respect to the surface (and make it appear as a short cell with |*A*| ≈ 0). Therefore, cells with |*A*| ≈ 0.5 are not present amongst the selection of longer cells. These effect would not be present in 3D time-series.

Interestingly, the data reveals that actively rotating cells (Fig 6e) exhibit an increased probability of polar adhesion (compared to pivoters, Fig 6d), probably because a fraction of rotators is adhering with a short filament or part attached to the flagella motor and the concentration of flagellar motors is highest at the poles [30].

As was discussed before [9], wobbling cells adhere to the surface with multiple adhesive patches. Therefore, they are parallel to the surface, and the projection artefacts observed for pivoters and rotators are not an issue. We can calculate a distribution of the expected anchoring point for wobblers (gray lines in Fig 6f) by placing *n* imaginary adhesive patches along the length axis using the measured *P*(*A*) for pivoters. For this dataset, we find a good match with the experimentally observed distribution (blue line in Fig 6f) for *n* = 2, 3, as was found before [9].

### Precision in measuring the anchoring point

**Fig 7:**
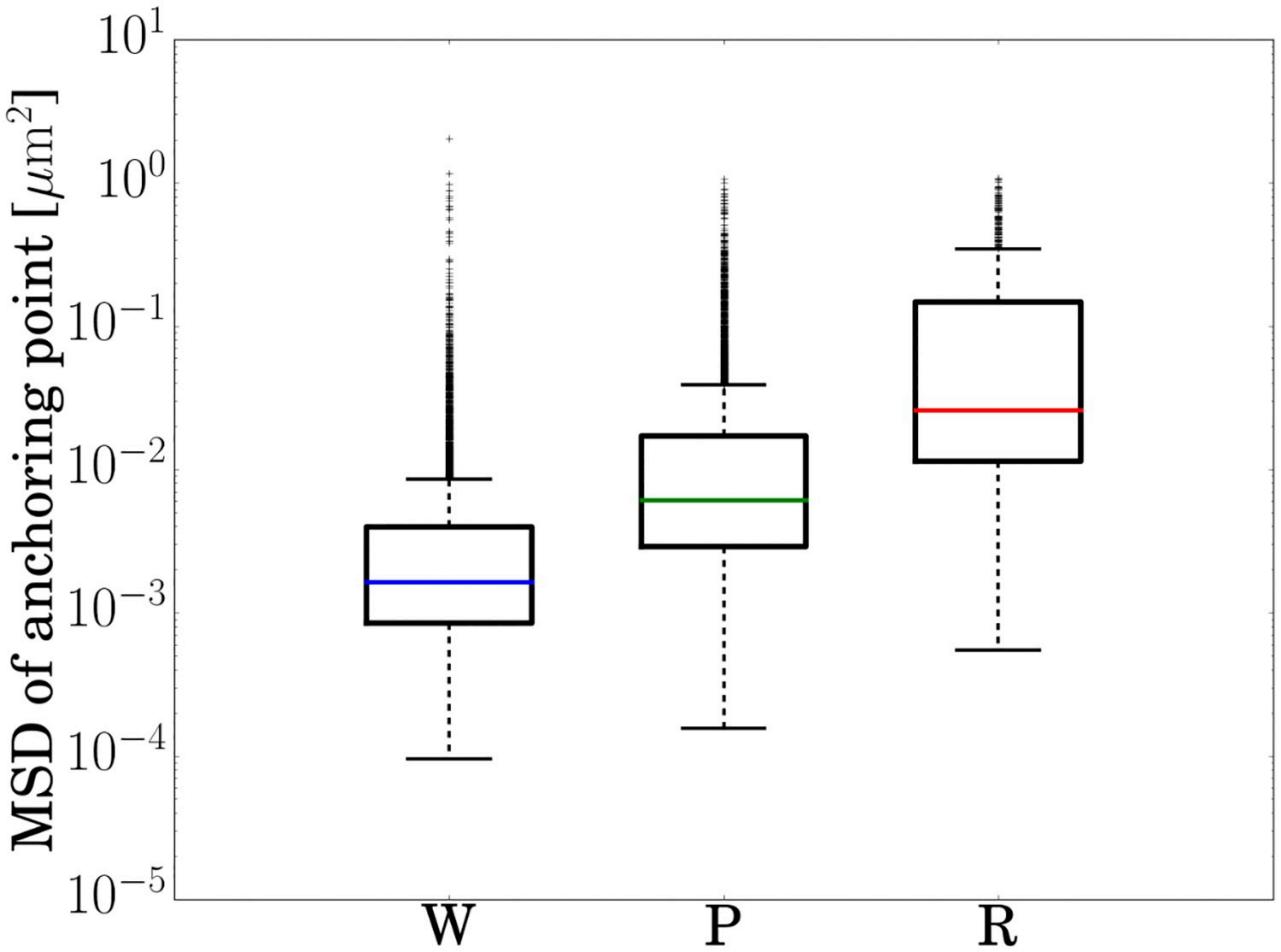
Box-plots of the anchoring point MSD for wobbling, pivoting and rotating cells, measured over a delay time *τ* = 0.4s. As wobbling cells are firmly stuck to the surface, their MSD gives an upper bound for the measurement precision. The box contains data in the inter-quartile range (IQR) between quartiles Q3-Q1. The average (mean) is at the middle of the box. Coloured horizontal lines give median values. Upper whiskers have range 1.5×IQR from the box, lower whiskers are minimum data values. Individual marks are outliers.

To estimate the precision with which the anchoring point is determined, we analyse the mean-square-displacement of adhering cells (Fig 7). Of these, wobblers are the most firmly attached cells, for which we obtain a median MSD ≈ 0.002 μm^2^. This corresponds to a 0.04 μm root-mean-squared displacement between frames, serving as an upper-bound on the error in the displacement of tracked rods between consecutive frames.

### How a systematic error in the MSD affects the translational exponent of adhering cells

**Fig 8:**
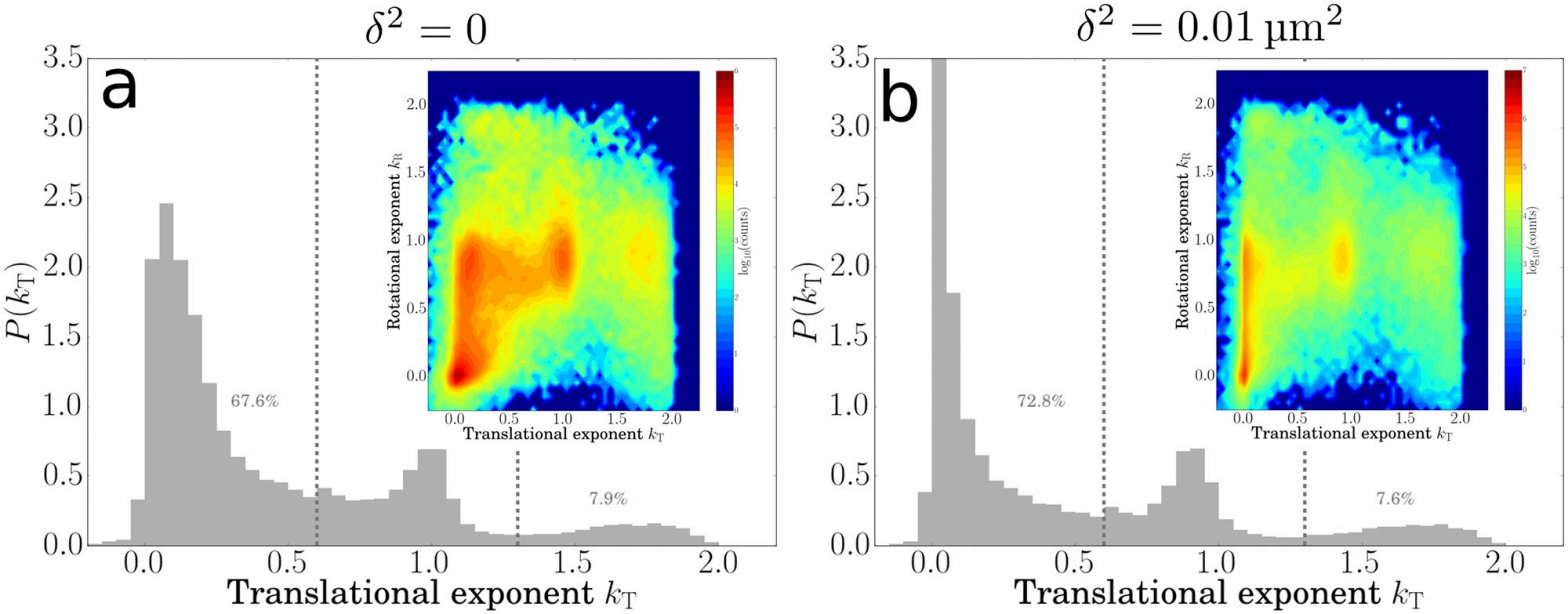
How systematic errors affect the exponent landscape. Histograms of translational exponents $k_{T}^{0.4s}$ for bacteria on the surface, **(a)** as measured (*δ*^2^ = 0), and **(b)** with a systematic error *δ*^2^ = 0.01 μm^2^ added to the MSD. Insets show 2D corresponding histograms for translational and rotational exponents. Note the shift in the position of the peaks for *k*_T_, while the position in *k*_R_ remains unaffected.

The translational exponent *k*_T_ of adhering cells depends on the time-scale over which it is measured. Over short time scales, even the most firmly adhered cells show some diffusive motion and *k*_T_ will shift from zero towards one, whereas over long time scales the mean-squared displacement saturates for adherers so that *k*_T_ → 0. Moreover, *k*_T_ also depends non-trivially on the precision with which the position of the anchoring point is determined. Fig 8a shows the raw distribution of the translational exponent, with peaks for adhering, diffusing and swimming cells. Fig 8b shows the effect of introducing a random error in all positions by adding 0.01 μm^2^ systematically to all the MSDs. This *δ*^2^ is higher than the MSD of adhering cells, but lower than the MSD of diffusing cells, causing the peaks for adhering cells to shift toward lower exponents (Fig 8b). The two-dimensional representations corresponding to the two distributions are shown in the insets to Fig 8a and 8b. It is clear that for pivoting cells the shift toward lower exponents ∼ 0 is particularly dramatic. This effect can be understood analytically by writing the exponent in Eq 1 as

$$\begin{array}{c} \begin{array}{cc} k_{T}= & \frac{\text{log}\left(\langle R^{2}\left(\tau_{1}\right)\rangle +\delta^{2}\right)-\text{log}\left(\langle R^{2}\left(\tau_{0}\right)\rangle +\delta^{2}\right)}{\text{log}\left(\tau_{1}\right)-\text{log}\left(\tau_{0}\right)}=\frac{\text{log}\frac{\langle R^{2}\left(\tau_{1}\right)\rangle +\delta^{2}}{\langle R^{2}\left(\tau_{0}\right)\rangle +\delta^{2}}}{\text{log}(\frac{\tau_{1}}{\tau_{0}})}\\ & =\frac{\text{log}\frac{\langle R^{2}(\tau_{1})\rangle }{\langle R^{2}(\tau_{0})\rangle }}{\text{log}(\frac{\tau_{1}}{\tau_{0}})}+\frac{\text{log}\frac{\langle R^{2}(\tau_{1})\rangle +\delta^{2}}{\langle R^{2}(\tau_{1})\rangle }}{\text{log}(\frac{\tau_{1}}{\tau_{0}})}-\frac{\text{log}\frac{\langle R^{2}(\tau_{0})\rangle +\delta^{2}}{\langle R^{2}(\tau_{0})\rangle }}{\text{log}(\frac{\tau_{1}}{\tau_{0}})}, \end{array} \end{array}$$ (3)

where the negative term on the right becomes large as *δ*^2^ becomes of similar order as *R*^2^(*τ*_0_), causing the translational exponent tends to shift to shift to smaller values upon increasing *δ*^2^. This result illustrates the importance of accurately determining bacterial positions before performing detailed further analysis. In particular, one should be aware that even random errors occurring at a frame-to-frame timescale can obscure longer-time dynamical information.

### How the translational exponent distribution for diffusing cells depends on trajectory durations

In our experiments, we classify different types of motion via the translational exponent *k*_T_, whose value should be 1 for an infinitely long trajectory of a freely diffusing cell. In experiments, however, diffusing cells can move in and out of the observation plane. As a result, each trajectory has a finite duration, which will produce a distribution of *k*_T_ values. To validate our experimental analysis, we compared the experimental *k*_T_ distributions with simulations of diffusing cells and an approximate, analytic theory.

To calculate an approximate expression for the probability distribution *P*(*k*_T_) for diffusing cells, we start from the probability density function for 2D diffusion away from the origin

$$\begin{array}{c} P\left(r;\tau \right)=\frac{1}{4\pi D\tau }e^{\frac{-r^{2}}{4D\tau }}\;, \end{array}$$ (4)

where *r* = |**r**| is the distance from the origin. By integrating over the polar angle and making the substitution *U* = *r*^2^ we obtain

$$\begin{array}{c} P\left(U;\tau \right)=\frac{1}{4D\tau }e^{\frac{-U}{4D\tau }}\;. \end{array}$$ (5)

with the expectation value for $\text{MSD}(\tau )=\int_{0}^{\infty }Up\left(U\right)dU=4D\tau$.

In practice it is possible to calculate the MSD in several ways. For the analysis of experimental data in this paper, we average over every possible pair of frames separated by the delay time *τ*, which makes the most complete use of the data. However, because the video segments spanned by these pairs of frames overlap, they are not independent, so it is difficult to obtain an analytical prediction for this case. Thus, for the purpose of this calculation we consider instead the MSD obtained by averaging over independent, non-overlapping segments. This difference affects the final width of the distribution, as we discuss below. We split each trajectory of duration *T* into *β* = *T*/*τ* independent segments. We define ${\bar{U}}_{\beta }\left(\tau \right)$ as the MSD obtained by averaging over those segments for a single trajectory, i.e.,

$$\begin{array}{c} {\bar{U}}_{\beta }\left(\tau \right)=\beta^{-1}\sum_{i=1}^{\beta }{\left|r(i\tau )-r((i-1)\tau )\right|}^{2}\;. \end{array}$$ (6)

Then the probability distribution of ${\bar{U}}_{\beta }\left(\tau \right)$ is given by the probability distribution for the mean of *β* individual selections from the exponential probability distribution given by Eq (5), which results in the so-called Erlang distribution

$$\begin{array}{c} P\left({\bar{U}}_{\beta }\left(\tau \right)=x\right)={\left(\frac{\beta x}{4D\tau }\right)}^{\beta }\frac{e^{-\frac{\beta x}{4D\tau }}}{x\Gamma \left(\beta \right)}, \end{array}$$ (7)

where Γ is the gamma function. The exponent *k*_T_ is defined by

$$\begin{array}{c} k_{T}=\frac{\ln({\bar{U}}_{\beta }\left(\tau \right)/{\bar{U}}_{\alpha \beta }\left(\tau_{0}\right))}{\ln\tau /\tau_{0}}\;, \end{array}$$ (8)

where *τ*_0_ = 1/framerate is the first delay time in the window from which *k*_T_ is extracted and *α* = *τ*/*τ*_0_. In principle this calculation involves two distributions over the MSD, at delay times *τ*_0_ and *τ*. Instead, we make the approximation of a high frame rate compared to the delay time, i.e., *α* ≫ 1, which allows us to replace the probability distribution at *τ*_0_ with a delta function, ${\bar{U}}_{\alpha \beta }\left(\tau_{0}\right)=4D\tau_{0}=4D\tau /\alpha$ because this distribution is over *αβ* repeats, and will therefore be much narrower than the distribution at *τ*, taken over *β* repeats only. With this approximation, we obtain finally

$$\begin{array}{c} P\left(k_{T}\right)=\frac{\ln\alpha }{\Gamma (\beta )}{\left(\beta \alpha^{k_{T}-1}\right)}^{\beta }\exp(-\beta \alpha^{k_{T}-1})\;. \end{array}$$ (9)

**Fig 9:**
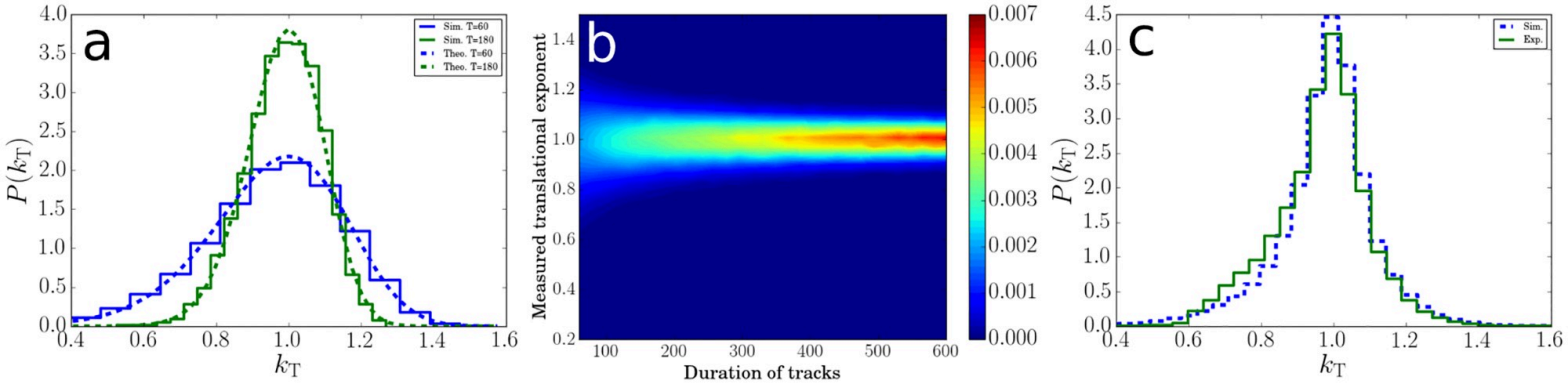
Distribution of the translational exponent for trajectories of diffusing cells. **(a)**, Probability distribution of the exponent *k*_T_ for diffusers, from simulated tracks of specific duration (*T* = 60 and *T* = 180 frames), compared with the theoretical result (Eq 9). The mean-squared displacements arising from the theoretical model assume calculations with non-overlapping time segments. To compare with theory (Eq 9), the simulation results in this figure have been calculated in the same way. **(b)**, Contour plot of probability to measure a certain translational exponent in simulations for different durations of a trajectory calculated using overlapping segments. Data is normalised such that the sum of probabilities for each trajectory duration (column) adds up to one. **(c)**, Probability distribution of the exponent *k*_T_ for diffusers, from simulated tracks and from experiments for ΔFF and 0.2w% TWEEN 20. The simulated trajectories have the same durations as the trajectories from the experiments. The MSD from simulations in this figure have been calculated based on averages over overlapping segments, exactly as in the experiments.

The distribution *P*(*k*_T_) from Eq 9 is plotted in Fig 9a for two different track durations (dashed lines), showing that as the track duration is decreased, it becomes broader while showing an increased skewness towards lower exponents. The translational exponents can also be calculated from computer simulations. In these simulations, non-interacting particles are allowed to freely diffuse for a duration *T*, with the displacement **r** = {*x*_1_, *x*_2_} of each particle calculated using

$$\begin{array}{c} x_{\xi }\left(t+\Delta t\right)=x_{\xi }\left(t\right)+\zeta_{\xi }\left(t\right)\sqrt{2D\Delta t}, \end{array}$$ (10)

where *ξ* = 1, 2 indexes the dimension, the *ζ*_*ξ*_(*t*) are random stochastic variables drawn from a normal distribution centred at zero with unity standard deviation, *D* is the diffusion coefficient, and *Δt* is the time interval between consecutive timesteps. After this the exponent is calculated from the MSD by using non-overlapping segments as defined in Eq (6), and the distribution of exponents is plotted in Fig 9a, showing good agreement with the theoretical prediction. We also calculated the simulated *k*_T_ distribution by using overlapping segments to calculate the MSD (red curve). The resulting distribution has a similar shape but is narrower than the equivalent distribution calculated with non-overlapping segments. This is expected since using overlapping segments makes complete use of the data, so should provide a more precise estimate of *k*_T_.

We also investigated systematically how the distribution of *k*_T_ with non-overlapping segments depends on the trajectory duration by running a series of simulations, each using a different duration that was fixed for all trajectories within that simulation. Fig 9b shows how the distribution of *k*_T_ narrows for increasing trajectory duration and approaches 1 for long trajectories. The choice of *D* and Δ*t* in the simulations did not affect the distribution of *k*_T_.

We also compared the exponents calculated from trajectories in the computer simulations with those calculated from experimental trajectories of diffusing cells. To make sure that there were no swimmers in the population, we used a strain of nonflagellated bacteria (ΔFF_MG_). To minimise the number of adhering cells on the surface, we performed the experiment using a buffer solution containing 0.2w% of TWEEN 20, which effectively blocks the adhesion of almost all of the bacteria to the surface [10]. For the simulations, we used exactly the same distribution of trajectory durations as in the experimental dataset, and now used overlapping segments to calculate the MSD, as in the experiments. Again, we find good agreement, Fig 9c. This validates our data analysis technique, and demonstrates that, at least for diffusing particles, we can safely neglect optical artefacts and selection effects (e.g., the depth of focus is limited, so that there will be a coupling between diffusivity *D* and trajectory duration *T*).

## Summary

The automated analysis and interpretation of microscopy images containing microorganisms is of academic and practical relevance. Studying bacteria on the level of single cells has the potential to reveal new important phenomena related to bacterial adhesion and population heterogeneities [9, 20, 31–35]. Therefore, algorithms that can extract this information from videos are highly relevant to study differences between individual cells within a population, which has potential applications in developing medical diagnostics methods.

We have previously described [9] variations in adhesion properties of cells within a clonal population. Our conclusions were based on a detailed analysis of microscopy videos. In this work, we have discussed in detail the algorithms used to identify, follow and characterise bacteria on the surface from images in recorded microscopy movies using a dataset similar to [9] but for a different batch of freshly prepared bacteria. To aid the future development of analysis software for the high-throughput analysis of microscopy movies in experiments, we make available this data as well as the source code (mainly written in the programming language *C* for Linux) to find, track and analyse the dynamics of rod-shaped bacteria [13–18].

We have discussed algorithms to determine the positions and orientations of individual bacteria (*findRods2Dt*) and subsequently calculate and filter their trajectories in time (*trackRods2Dt* and *filterTracks2Dt*). We explained in detail how the mean-square displacement (MSD) of the least mobile point on each cell is then used to calculate a translational exponent to characterise the dynamics of individual cells (*analyzeBugTracks2Dt*). For adhering cells, we explained how the rotational exponent from the orientational mean-square displacement is used to characterise post-adhesion dynamics.

To validate our results, we have performed a quantitative analysis of how errors and limited trajectory durations affect the translational exponents of adhering and diffusing cells. We found that exponents of adhering cells shift towards zero upon addition of a random error in particle position. We also provided a theoretical description of how the skewed distribution of translational exponents for diffusing cells depends on the duration of the trajectories. For long trajectories, the distribution is strongly peaked around 1, but broadens for shorter trajectories. We verified the theory with simulations and experiments.
